# Behavior and Speech Features of Children with ADHD

**DOI:** 10.3390/healthcare14060814

**Published:** 2026-03-22

**Authors:** Elena Lyakso, Olga Frolova, Andrey Lebedev, Petr Shabanov, Severin Grechanyi, Elina Atamanova, Marina Kovelenova, Victoria Limarenko

**Affiliations:** 1 Child Speech Research Group, Department of Higher Nervous Activity and Psychophysiology, Biology Faculty, St. Petersburg State University, 7-9 Universitetskaya Embankment, 199034 St. Petersburg, Russia; olchel@yandex.ru (O.F.); svgrechany@mail.ru (S.G.); 2 “Doctrina” Clinical Medical Center, Kolomyazhsky Prospect, 33, 197341 St. Petersburg, Russia

**Keywords:** attention deficit/hyperactivity disorder, behavior, speech features, co-op play, children

## Abstract

**Background/Objectives**: The goal of the study was to identify the peculiarities of attention deficit hyperactivity disorder (ADHD) on the base of the behavioral characteristics and acoustic features of speech of children with ADHD and ADHD with comorbidity—ADHD and autism spectrum disorders (ASD) and ADHD and intellectual disabilities (ID)—within the framework of one test task. Behavioral characteristics were selected using DSM-V criteria; acoustic features of speech were considered by researchers as speech markers of ASD and ID detected for different languages. **Methods**: The study includes 92 children aged 5–13 years with ADHD, ADHD and ID, ADHD and ASD, and control groups of children diagnosed with ASD, ID and typically developing (TD) children. The children were tested using the test task “co-op play”. Video and audio recordings of children performing the test task were collected. We used a complex approach to study the peculiarities of children with ADHD through expert analysis of children’s behavior and play, acoustic spectrographic analysis of speech and questionnaires about early childhood development filled out by parents. **Results**: The characteristics of behavior, play, and acoustic features of speech of children with ADHD and ADHD and comorbidity were revealed. Children with ADHD had lower behavior scores in the play situation on the expert assessment than TD children, with the greatest differences for characteristics of play, “Playing for toy”, and of behavior “Displaced activity” and “Losing attention”. The speech of children with ADHD is characterized by low values of the third formant and the difference between the first two formants, compared to the corresponding speech features of children from other groups. The speech of children with ADHD+ASD is characterized by maximal pitch values (high voice), while that of children with ADHD+ID is characterized by low vowel articulation index values. **Conclusions**: Based on the analysis of behavior and speech of children with TD, ADHD, ADHD and comorbidity performing the “co-op play” test task, the set of characteristics specific to ADHD was identified. The obtained data expand our understanding of the specificity of children with ADHD and may contribute to the development of qualified support for families with children with ADHD.

## 1. Introduction

Attention deficit/hyperactivity disorder (ADHD) is a type of neuropsychiatric development caused by interactions between genetic and environmental factors that has a worldwide prevalence of 5–15% in children [1] or of 5–7% according to the Diagnostic and Statistical Manual of Mental Disorders 5th edition (DSM, V) [2]. In the DSM IV [3], ADHD was combined into one disorder with three subtypes: predominantly inattentive, predominantly hyperactive, or combined type. The diagnostic indications for ADHD given by the DSM-V [2] include descriptions of nine symptoms in each of two domains (inattention and hyperactivity/impulsivity). The symptoms begin at a young age and usually include lack of attention, lack of concentration, disorganization, difficulty completing tasks, forgetfulness, and losing things [4]. To be diagnosed using these criteria, six or more symptoms and signs from one or each group must be present, and the symptoms must have appeared for six or more months with an onset before the age of seven [5]. Hyperactivity, attention deficit, and anxiety are often symptoms of various neurological and psychiatric diseases. Comorbidity is a distinct clinical feature of both children and adults with ADHD [6]. ADHD has a high comorbidity with other developmental disorders such as autism spectrum disorder (ASD) [7], intellectual disability (ID) and other mental illnesses.

A total of 65–80% of patients with ADHD have conduct problems and other comorbidities, in addition to low academic achievement and poor social and organizational skills [8]. It is emphasized that comorbid neurodevelopmental, behavioral, and emotional disorders are the rule rather than the exception [9]. Common signs include difficulty completing tasks, losing things, constant movement, fidgeting, difficulty staying still, and difficulty adjusting their behavior to changing situations [10]. ADHD is accompanied by speech disorders [11,12], emotional dysregulation [13,14], and social, school skills and behavioral disorders [15].

Children with ADHD are characterized by high levels of defiant, disruptive and intrusive behavior, poor peer interactions, and impaired interpretation of social situations [16]. According to the DSM-V, children and adolescents with ADHD are characterized by behavior peculiarities manifested in inattention, hyperactivity, and impulsivity [2]. Play activities are considered a tool for developing social play skills in children with ADHD [17,18]. “Co-op play” can serve to demonstrate the main behavioral manifestations of ADHD. Inattention is characterized by children making many careless mistakes while completing tasks, often failing to pay close attention to details, finding it difficult to concentrate on one thing for a long time while playing or doing something else, and frequently being distracted by their surroundings. Hyperactivity manifests itself as frequent restlessness; children fidget with their arms or legs, fidget in their chairs, and are constantly on the move. Impulsivity can manifest itself as disrupting turn-taking.

At the same time, the insufficient number of studies on children’s behavior in play situations allows us to formulate one of the study objectives and the first hypothesis that behavioral characteristics will be evident in “co-op play” and will differ in children with ADHD and their TD peers.

Children and adults with ADHD may have speech disorders at all levels of their organization and peculiarities of speech perception. Speech features can be adapted to delayed speech development, insufficient formation of articulations, and voice disorders [11,19].

Children with ADHD are more talkative [20], and more likely to speak faster, be hoarse [21], and have a louder voice [20,21] than their typically developing peers. The study of pitch values of children with ADHD is rare and significant differences between ADHD and TD children were not revealed [22,23].

Parent ratings of 188 children (average age 4 years 7.5 months) who completed the Strengths and Difficulties Questionnaire [24] and the Speech and Language Difficulties Questionnaire found that children with ADHD were significantly more likely to experience speech and language problems than peers without ADHD [25]. A questionnaire survey of 300 children showed that in children with severe ADHD, there was a significant correlation between speech sound disorder and ADHD, and a significant correlation between childhood apraxia and ADHD [12]. Another study showed that approximately 30% of children with ADHD experience language delays [26]. Approximately 30% of children with ADHD exhibit significant delays in reading development, and approximately 40% of these children experience difficulties with phonological processing [27,28], which impacts their reading and writing skills [29]. Phonological problems are associated with difficulties with articulation and accurate phoneme identification, as well as with the rhythmic organization of words, leading to mispronunciation, suggesting broader auditory processing and working memory deficits [30].

Pragmatic [26,31,32] and semantic [26] language disorders and, to a lesser extent, phonetic-phonological aspects [26] are common in children with ADHD. Data from another study of the speech development of 30 Egyptian Arabic-speaking 4- to 7-year-old children with ADHD confirmed that most children had problems with pragmatic aspects of speech, which correlated positively with ADHD symptoms [33]. Using the Preschool Language Development Scale 4th Edition (Arabic version), the Articulation Test in Arabic, and the Egyptian Pragmatic Language Test, children with ADHD participating in the study demonstrated moderate delay in speech development compared to children in the control group. However, 50% of children with ADHD demonstrated delays in acquiring sounds corresponding to their phonological age [33].

The influence of speech comprehension skills and behavioral characteristics on school readiness in 49 preschool-aged children with ADHD was found. Behavioral manifestations of ADHD are a risk factor in the relationship between speech comprehension deficits and social-emotional school readiness [15]. Other researchers showed that ADHD is not accompanied by language delays in the use of syntax and semantics, but there is a significant link between ADHD and communication disorders, since speech disorders are secondary [34]. Thus, based on various approaches, questionnaires, and scales, the characteristics of speech and language development in children with ADHD are demonstrated. The degree of their severity is largely determined by the method being used. One of the universal approaches may be an instrumental analysis of the acoustic features of speech in children with ADHD, since it allows us to identify voice and speech features that are independent of the language and cultural environment in which the child is raised. Such studies are rare [21,22,23].

In various studies [35,36,37,38,39,40,41,42,43] devoted to identifying speech markers of diseases based on the acoustic features of speech, the main ones considered are the pitch values (voice pitch) and articulation characteristics—the values of formants and the vowel articulation index, which determine the clarity of articulation and affect speech intelligibility. On the one hand, given the paucity of such data for ADHD and their absence in the Russian language, and on the other hand, given the possibility of using acoustic characteristics of speech as markers of the disease, we formulated a research hypothesis.

Children with ADHD will differ from TD children in articulatory characteristics—formant values and from children with ADHD and comorbidity in pitch values and articulation clarity.

Thus, the study tests two hypotheses about the differences between children with ADHD and TD in behavioral characteristics and in the acoustic features of speech.

The goal of the study was to identify the peculiarities of ADHD on the basis of behavioral characteristics and acoustic features of speech of children with ADHD and ADHD with comorbidity—ADHD and ASD and ADHD and ID—within the framework of one test task.

### Research Questions

The present study was guided by the following research questions:

First, we analyzed medical records to recruit children with ADHD and ADHD with comorbidity for the study. Using early development questionnaires completed by their parents, we identified early developmental indicators that predicted subsequent diagnosis.

Second, we identified behavioral characteristics of children during “co-op play” that were specific to children with ADHD, ADHD with comorbidity, and differed from those of TD children.

Third, we analyzed the acoustic features of speech in children across all study groups while performing the “co-op play” test task. We identified voice and speech characteristics specific to ADHD and ADHD with comorbidity—ADHD+ASD, ADHD+ID.

Fourth, we assessed the influence of children’s age on all analyzed acoustic features.

Fifth, we further examined whether the severity of autism spectrum disorders (measured by the CARS scale) influences the acoustic features of speech in children with ASD and ADHD+ASD.

We summarized the data on the characteristics of behavior and speech of children with ADHD.

## 2. Materials and Methods

### 2.1. The Study Design

Using a complex approach, including non-invasive methods of recording and analysis of children’s behavior and speech, we directed our efforts to identifying specific characteristics of ADHD. For this purpose, we developed criteria for inclusion of children in the study, selected children with ADHD, ADHD with comorbidity, and control groups for children with ADHD and ADHD with comorbidity, and TD children—controls for children in all groups—and tested the children using the standardized method. The children’s behavior and speech were recorded when performing the “co-op play” test task. This test task is standardized and well-suited for collecting material for TD children and children with developmental disabilities, allowing us to identify the specifics of the behavior and speech of children, which is difficult to achieve using only interviews [44].

This study tests the hypothesis that children with ADHD differ from TD children and children with comorbidity in behavioral characteristics identified through “co-op play” and acoustic speech features. Behavioral characteristics were selected based on the DSM-V criteria, and acoustic features were considered by researchers as markers of ASD, Down syndrome (DS), and ID for different languages [35,36,37,38,39,40,41,42].

To estimate the frequency of different forms of ADHD, the distribution of children with ADHD by gender, and the selection of children for study, the medical documentation of 1568 children who attended the medical center were analyzed.

Data analysis included an expert analysis of children’s behavior during “co-op play” (Study 1) and an instrumental analysis of the acoustic features of children’s speech (Study 2). This approach allowed us to identify behavioral characteristics and acoustic features of children’s speech specific to the disorder.

The study was conducted in accordance with the Declaration of Helsinki. The parents of the children participating in the study signed an informed consent approved by the Ethics Committee of St. Petersburg State University—protocol N 115-02-101.

### 2.2. Analysis of Medical Documentation

To estimate the frequency of different forms of ADHD, the medical documentation of 1568 children who attended the medical center were analyzed. The analysis of children’s medical documentation was conducted using the existing medical database with coded names of children in the “Doctrina” Clinical Medical Center in December 2026. The criteria for selecting the relevant medical documents are the period from 1 January 2024 to 15 December 2025 and the presence of an ADHD diagnosis in the medical documents.

Additionally, we reviewed medical documentation in which the child had the following symptoms: hyperactivity, impulsivity, attention deficit, and impaired school performance. The following information was extracted from the selected medical documents: date of examination, age and gender of the child, information about the family, the course of the mother’s pregnancy and childbirth, anamnesis vitae and morbi, and diagnosis. Analysis of this information was necessary to detect the frequency of ADHD without comorbidities and overlapping symptoms and to identify the most significant indicators of children’s early development that influence their diagnosis.

The analysis of medical documentation showed that among all children who attended the medical center over the two years, 95 children (6.1%) had an ADHD diagnosis in the medical documentation. We selected three groups according to the refined diagnosis: ADHD (19% of children with ADHD), ADHD+ID (70.5%), and ADHD+ASD (10.5%). In each group of children, boys predominated (Table 1).

Information from medical documentation allowed us to reveal a correlation between the group of children and the characteristics of early development of children, the age of the first words, and their speech development (normal/abnormal) (Table 2). To apply regression analysis to categorical data, the groups of children were designated by numbers: 1—ADHD, 2—ADHD+ID, 3—ADHD+ASD. This analysis was an additional confirmation of the need to study the speech development of children with ADHD because speech development and the age of first words could be considered as predictors of comorbidities in ADHD. Correction for multiple comparisons (Bonferroni test) showed the significance of all identified parameters.

### 2.3. Participants of the Study

#### 2.3.1. Recruitment Procedures and Recruitment Channels

Children with ADHD, ADHD with comorbidity (ADHD+ASD, ADHD+ID), and control groups (ASD, ID) attending the Medical Center and matching the inclusion criteria were invited by medical doctors to participate in the study for testing from 19 June 2025 to 15 December 2025. TD children were recruited by posting the information about the study in kindergartens and schools, which were the scientific bases for studies of St. Petersburg State University. TD children were selected by interviews with the child, their parent, and parent completion of the child development questionnaire, considering inclusion/exclusion criteria. All TD children included in the study were not diagnosed with developmental disabilities.

#### 2.3.2. The Criteria of Inclusion

For all children: Age of children was 5–13 years; informed consent was approved by the Ethics Committee of St. Petersburg State University for the child’s participation in the study, signed by parents.

For children from the TD group: 1. the absence of prenatal development risks diagnosed by neonatologists; 2. Apgar scores were not lower than 6/7 points; 3. no serious visual or hearing impairments.

For children in the ADHD, ADHD+ASD, and ADHD+ID groups:

1. Presence of the confirmed diagnosis. Children with ADHD+ASD and ADHD+ID had comorbidity. The comorbidity was determined based on the DSM-V diagnostic interview. If additional symptoms (ASD and ID) met the diagnostic criteria for a specific disorder, the comorbidity (ADHD+ASD, ADHD+ID) was diagnosed. All diagnoses were established by board-certified pediatric neurologists and child psychiatrists strictly following DSM-5 criteria, with alternative etiologies (trauma, epilepsy) excluded. Core ADHD symptoms were assessed using multi-informant clinical interviews. Symptoms extending beyond core DSM-5 boundaries, such as emotional dysregulation, were evaluated using the adapted Strengths and Difficulties Questionnaire (SDQ). To operationalize the distinction between true comorbidity and symptom overlap, clinicians apply two approaches. First, a subtraction method was used, where shared symptoms (e.g., impulsivity in Oppositional Defiant Disorder) were discounted to ensure the secondary diagnosis independently met threshold criteria. Second, a chronological method was applied based on developmental history: early-onset emotional dysregulation was classified as an ADHD phenotypic feature, while late-onset emotional disturbances were evaluated as distinct comorbid disorders. 2. The children’s speech development level allowed using words and simple phrases.

The children’s parents filled out questionnaires using the Child Emotional Development Method (CEDM) [44] about the mother’s pregnancy and her health, the early and current development of the children, and their behavior.

#### 2.3.3. The Criteria of Exclusion

1. The absence of informed consent signed by parents. 2. If symptoms additional to ADHD symptoms did not fit within the diagnostic criteria, then the symptoms were considered as combined disorders (overlapping symptoms) and the child was not included in the groups with comorbidity—ADHD+ASD and ADHD+ID—and was not included in the study. 3. For children with ADHD+ASD and ASD—a severe form of autistic symptoms—scores on the CARS scale are 44–60 [45]. Parents of children with ASD and ID completed the CARS scale because autistic disorders may accompany intellectual disabilities as concomitant symptoms. For children with ID—mild to moderate impairment. 4. Children with ADHD+ ID, with a primary diagnosis of Down syndrome, cerebral palsy, and children with severe and profound intellectual disabilities.

#### 2.3.4. Groups of Children

The participants of the study were 92 children aged 5–13 years and 6 experts with professional experience (professional experience—16 ± 6 years). Based on the diagnosis, children were divided into six groups: ADHD children, children with ADHD+ASD, ASD, ADHD+ID, ID, and TD children. The group of TD children is the control for all groups, ASD is the control for ADHD+ASD, and ID is the control for ADHD+ID.

According to the aim of the study, two studies were conducted: 92 children took part in the first study and 60 children in the second study (Table 3).

The sample of children in the groups with ADHD, ADHD+ASD, and ADHD+ID (36 children) corresponds to the data obtained from the analysis of medical documentation (95 children) for the period from 1 January 2024 to 15 December 2025 by distribution and gender.

The scores on the questionnaire scales and the main characteristics of the early development of children in six groups are presented in the tables (Table 4).

The groups of children do not differ in their scores on the questionnaires for the “Gestation” and “Behavior and Health” scales. On the “Early Development” scale, children with ADHD, ADHD+ASD, and ASD have worse scores than TD children. On the “Behavior & Health” scale, children of all groups had worse scores compared to TD children. The analysis of the characteristics of the “Early Development” scale was conducted. The age of first words in all groups of children with disorders is significantly higher than in TD children. The age of sitting without support, standing up, and walking is significantly higher in children with ADHD than in TD children. Children with ADHD+ASD stand up later, and children with ASD begin to walk later, compared to TD children.

Thus, the retrospective analysis of medical documentation and information about early development of children included in the study showed that characteristics of early speech development are a predictor of diagnosis. So, we concluded that it is necessary to study the characteristics of speech of children with the diagnosis.

### 2.4. Testing Procedure

“Co-op play”: We invited a child and an experimenter to play with three toys—a parrot, a bunny, and a chick—in a “Birthday” scenario. The child named the toys and chose the main character whose birthday party they would be attending. The game included an invitation to the party, gifts, a birthday cake, and a game. “Co-op play” with the “Birthday” situation is one of the test tasks of the CEDM methodology [44], tested on 560 Russian and 400 Indian children [46]. The test task contains a standardized protocol for the sequence of actions of the child, directed by the experimenter.

Video and audio recordings of children performing the test task “co-op play” of CEDM were made in a laboratory condition (St. Petersburg State University) and a Medical Center (“Doctrina” Clinical Medical Center, St. Petersburg). The recording duration of the test task “co-op play” for each child was 2 to 5 min. The recording was carried out in a room measuring 18 m^2^, without a special noise-absorbing wall covering the noise level, which did not exceed 20 dB. The research conditions in the laboratory and medical center are as close as possible.

The procedure for testing the children was recorded on a SONY HDR-CX560 video camera, Sony Corporation, Minato, Tokyo, Japan (maximum resolution 1920 × 1080 at 50 frames per second), which was located at a distance of 1 m from the child’s face. The recordings of the speech of children were made by the “Marantz PMD660”, Masimo, Shirakawa Audio Works for Marantz products, Osaka, Japan, recorder with an external omnidirectional microphone “SENNHEIZER e835S”, Sennheiser electronic GmbH & Co., KG., Wennebostel, Germany, with the following settings: the sampling rate was set to 16,000 Hz and the mono audio channel was used in all the recording sessions. The recording was carried out in rooms without special soundproofing. The microphone was set at a distance of 30–50 cm from the child’s face. Speech files were stored in Windows PCM format WAV, 48,000 Hz, 16 bits per sample; video files were in AVI format.

### 2.5. Data Analysis

#### 2.5.1. Behavior Analysis

The video analysis of children’s performance of the test task “co-op play” was made using two approaches. In the first, 2 experts rated on a Likert scale [47] according to the criteria of the CEDM methodology [44]. In the second, 4 other experts determined the characteristics of the child’s behavior using a questionnaire assessing the child’s play and behavior during “co-op play”. The total duration of video fragments analyzed by each expert was 6.4 h. The expert could view each video fragment one to three times.

In the first approach, a 4-point assessment was made of: 1. the child’s involvement in the play with toys and the experimenter; 2. the child’s ability to establish eye contact with the experimenter during joint play; 3. the child’s facial expression. Two experts independently assessed these three indicators on a 4-point Likert scale: “1 = none, 2 = slightly, 3 = moderate, 4 = perfect”. The maximum number of points a child could receive was 12 points.

In the second approach, 4 experts determined the characteristics of behavioral elements for children from 6 groups (Instruction for experts, the Supplementary Materials). For this purpose, a questionnaire was developed, including 12 points, 6 points characterizing the child’s play and 6 points characterizing the behavior demonstrated by the child.

Play: 1. Included in the play. 2. Plays and talks for a toy. 3. Develops play, shows initiative. 4. Does the child follow the experimenter’s plan? 5. Is the child’s interaction with the experimenter successful? 6. Who determines the interaction—an adult, a child, together?

Child behavior: 1. Shows aimless motor activity: runs, jumps, tries to climb somewhere, often in unacceptable situations, spins, turns, restlessly moves arms or legs. 2. Gets distracted from the play. 3. Loses attention (freezes, withdraws into himself) during the game. 4. Answers questions without thinking, without understanding the meaning, often without listening to the question to the end. 5. Is the behavior emotional or not? 6. Do positive or negative emotions prevail?

Four experts independently assessed 12 indicators on a 4-point Likert scale. The criteria for scoring were: 1—no, throughout the entire recording “co-op play”; 2—less than half; 3—more than half; 4—completely.

#### 2.5.2. Spectrographic Analysis of Speech

Spectrographic analysis of speech was carried out in the “Cool Edit Pro” sound editor, based on the algorithms implemented in the Cool Edit Pro sound editor. The analyzed acoustic features reflect the main physiological processes occurring in the vocal tract during speech production. Temporal characteristics (vowel duration in the words) represent the processes of speech breath formation, the pitch values—the frequency of vocal fold vibrations—and the values of the first two formants—the articulation processes occurring in the oral cavity. The values of the first two formants (F1, F2) of vowels are acoustic keys for vowel identification. The third formant (F3) is essential for defining voice quality and distinguishing subtle vowel nuances. F3 is most sensitive to tongue tip position, lip rounding, and front vocal tract shape. F3 provides finer detail about the shape of the vocal tract [48].

For all speech samples, the duration (ms) of stressed vowels in words was determined by word/phrase, stress vowels: pitch values (F0)—average and of first three formants (F1, F2, F3) (Hz)—and intensity values E0 (dB). The vowel articulation index (VAI) [49] and the range between the first two formants [F2 − F1], as a more accurate characteristic reflecting the structure of the vocal tract, were calculated.

### 2.6. Statistics

Statistical analyses were performed using “STATISTICA-12.” The data was preliminarily checked for normal distribution (Distribution Fitting). We used non-parametric criteria: Spearman correlation (*p* < 0.05), Mann–Whitney test, regression analysis, and multiple regression analysis. To enable the use of regression analysis on categorical data, the groups of children were designated by numbers. For each case of the multiple regression analysis, the significance level *p* < 0.05 was used, for which correction for multiple comparisons was applied (Bonferroni test). The aim of the use of statistical analysis methods is to reveal correlations between behavioral characteristics in “co-op play” and groups of children; acoustic features of speech and groups of children in the age aspect. For this purpose, Spearman correlation analysis was used. The analyzed variables included groups of children, age, behavioral characteristics in role-playing games, and acoustic characteristics of speech. The Spearman correlations were validated by regression and multiple regression analysis. To determine differences between groups of children in the expert analysis of the video recording of the “co-op play”, the Mann–Whitney test was used. An agreement between two experts’ analysis of the video “co-op play” test task is assessed using the Cohen kappa statistic (k) [50]. Relative strength of agreement was associated with kappa statistics: slight (0.00–0.20), fair (0.21–0.40), moderate (0.41–0.60), substantial (0.61–0.80), and almost perfect (0.81–1.00) [51]. Agreement between 4 experts is analyzed by extending Cohen’s kappa–Fleiss’ kappa [52].

Multiple regression analysis was conducted to rank the contribution of behavioral characteristics in “co-op play” (independent or predictor variables) to a characteristic of a certain group (dependent variable). The aim of multiple regression analysis was to reveal the correlation between the group of children and the characteristics of early development of children in medical documentation; the characteristics of early development, scores for “Play” and “Behavior”, “Behavior & Health” and scores for “co-op play” by questionnaires; the scores for “Play” and “Behavior” by expert analysis.

Mann–Whitney U Test (*p* < 0.05) was used for comparing acoustic features of speech samples of children in 6 groups. The aim of regression analysis was to explore the correlation between the age of children and the acoustic features of speech. The effect size was calculated for the Mann–Whitney criterion to determine the practical significance of the differences.

## 3. Results

### 3.1. Study 1: Characteristics of Behavior in “Co-Op Play”

Table 5 presents the correlation between the early development indicators revealed as significant (Mann–Whitney test, Table 4) for the children who participated in the study, the “co-op play” scores assigned by two experts with almost perfect agreement (k = 0.96) for the “co-op play”, and the group of children (1—ADHD+ASD, 2—ASD, 3—ADHD+ID, 4—ID, 5—ADHD, 6—TD). Early speech development indicators—the age of first words, total scores for the “Behavior & Health” scale, and “co-op play” scores are distinguishing characteristics between groups of children. Correction for multiple comparisons (Bonferroni test) showed the significance of all identified parameters.

**Figure 1:**
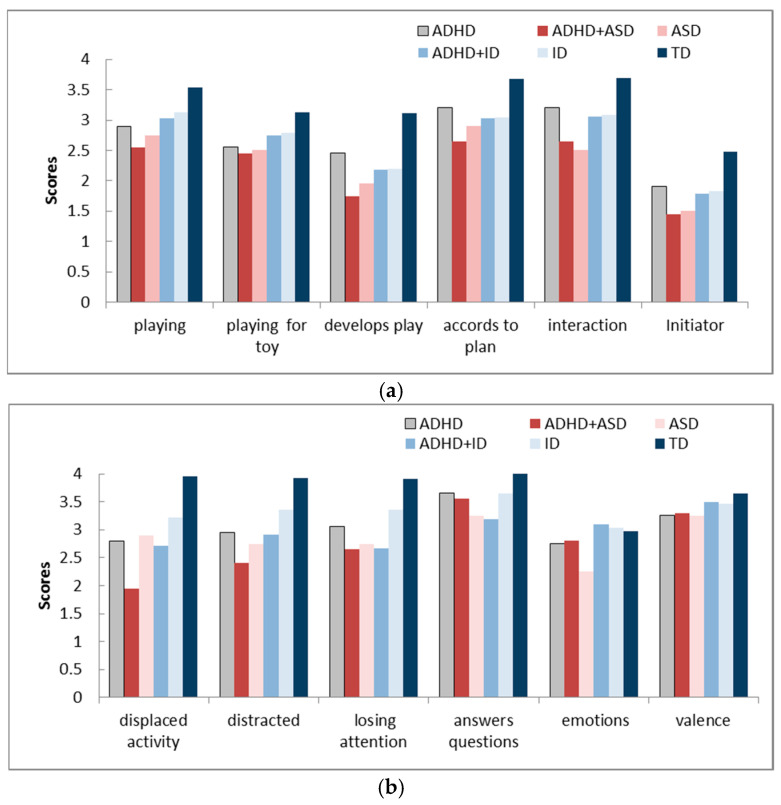
“Co-op play” characteristics—questionnaire scores (on a Likert scale) for “Play” (**a**) and “Behavior” (**b**) for children of 6 groups: data from responses of 4 experts (median values). Vertical axis—scores; horizontal axis—points characterizing the child’s “Play” (**a**), and the “Behavior” (**b**). Abbreviations: “playing”—included to the play; “playing for toy”—plays and talks for a toy; “develops play”—develops play, shows initiative; “accord to plan”—does the child follow the experimenter’s plan? “interaction”—is the child’s interaction with the experimenter successful? “initiator”—who determines the interaction—an adult, a child, together? (**a**); “displaced activity”—shows aimless motor activity: runs, jumps, tries to climb somewhere, often in unacceptable situations, spins, turns, restlessly moves arms or legs; “distracted”—the child gets distracted from the play; “losing attention”—during the game, the child loses attention (freezes, withdraws into himself); “answers questions”—answers questions without thinking, without understanding the meaning, often without listening to the question to the end; “emotions”—is the behavior emotional or not? “valence”—do positive or negative emotions prevail? (**b**).

Analysis of the video “co-op play” test task by four experts (the original data, the Supplementary Materials) showed significant differences in the “Play” and “Behavior” questionnaire points between the groups of children: ADHD and TD (*p* < 0.01; z = −2.598; r = −0.53, Mann–Whitney test); ADHD+ASD and TD (*p* < 0.001; z = −3.291; r = −0.672), ID (*p* < 0.05; z = −2.021; r = −0.412); ADHD+ID and TD (*p* < 0.01; z = −3.06; r = −0.625); ID and TD (*p* < 0.05; z = −2.425; r = −0.495); ASD and TD (*p* < 0.001; z = −3.349; r = −0.684); ASD and ID (*p* < 0.05; z = −2.078; r = −0.424) (Figure 1a,b).

Children with ADHD performed “Play” worse than TD children (*p* < 0.01); children with ADHD+ASD performed worse than children with TD (*p* < 0.001) and ID (*p* < 0.05); children with ADHD+ID performed worse than TD children (*p* < 0.01). Children with ID and ASD performed the task less successfully than TD children (*p* < 0.05 and *p* < 0.001, respectively); and children with ASD performed worse than children with ID (*p* < 0.05) (Figure 1a). Better agreement between experts was revealed for children with ADHD and comorbidity, ADHD+ASD and ADHD+ID, on characteristics of play—“Included to the play” (k = 0.48—moderate, k = 0.64—substantial) and “Plays and talks for a toy” (k = 0.43—moderate; k = 0.82—perfect).

According to the “Behavior” scores, the ADHD group has lower points than the TD group; the differences are the greatest for the “Displaced activity”, “Distracted from the play”, and “Losing attention” (Figure 1b). Minimal point scores were for children with ADHD and comorbidity—ADHD+ASD and ADHD+ID. Better agreement between experts was revealed on “Displaced activity” (k = 0.33—fair, for ADHD+ASD; k = 0.480—moderate, for ADHD+ID).

Table 6 presents data on the correlation between the group of children and the scores for “co-op play” characteristics. The characteristics that contribute most to classifying a child into a specific group were identified: overall scores for “co-op play”, “Develops play”, “Playing for toy”, “Losing attention”, and “Displaced activity”. These behavioral characteristics allow distinguishing the groups of children with ADHD and ADHD with comorbidity from the control groups. Correction for multiple comparisons (Bonferroni test) showed the significance of all identified parameters, with the exception of “Develops play”, at the trend level (*p* = 0.016, *p* < 0.0125).

The influence of age on the characteristics of child behavior was not revealed for the studied sample of children.

### 3.2. Study 2: Speech Features

**Figure 2:**
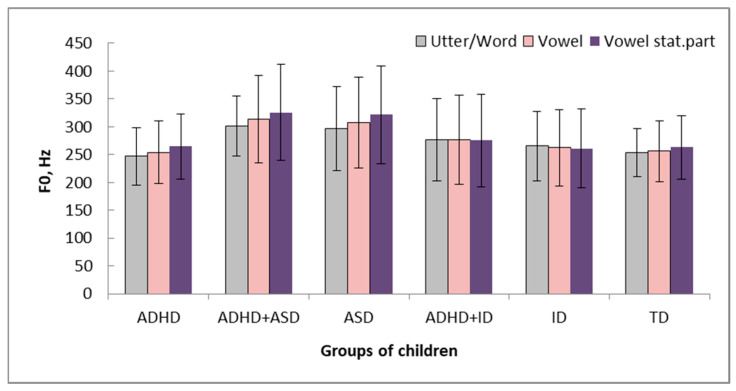
Pitch values of the utterances/words, vowels, and the stationary part of the stressed vowels for children from 5 groups (Average ± standard deviation). Vertical axis—pitch values (Hz), horizontal axis—groups of children.

Pitch values of the utterances/words, vowels, and the stationary part of the stressed vowels for TD children and children with ADHD do not differ significantly. Pitch values of children with ADHD differ significantly from pitch values of children with ADHD and comorbid disorders: on the utterances/words, stressed vowels, stationary part of the stressed vowels (Figure 2, Table 7).

The maximal pitch values for children with ADHD+ASD were revealed, which reflects the balanced influence of two comorbid disorders—ADHD and ASD. Thus, the study showed that the pitch values are informative diagnostic characteristics.

**Figure 3:**
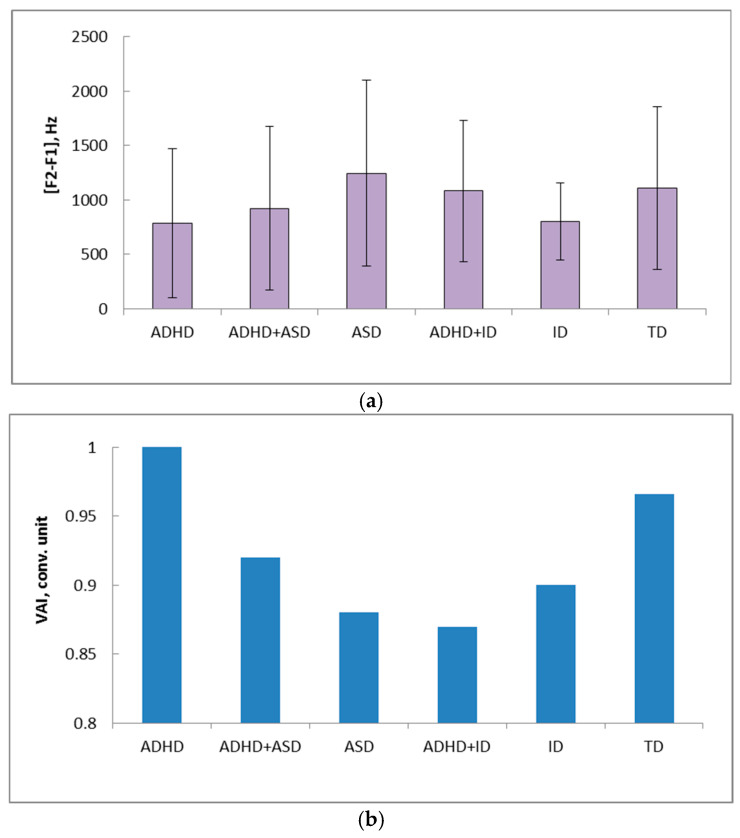
The [F2 − F1] values (**a**) and VAI (**b**) values for 6 groups of children. Vertical axis—[F2 − F1], Hz (**a**); VAI—conventional units (**b**); horizontal axis—groups of children (**a**,**b**).

The range values [F2 − F1] of stressed vowels are minimal (786 ± 686 Hz average ± standard deviation) in children with ADHD, and maximal in children with ASD (1245 ± 853 Hz). For ADHD children, the range values [F2 − F1] differ (*p* < 0.0001) from TD, ADHD+ASD, ASD, and ID children corresponding features (Figure 3, Table 6).

The VAI values are the highest in children with ADHD (1.0), with high VAI values in children of all groups, and with the lowest values in children with ADHD+ID (0.87) (Figure 3b).

**Figure 4:**
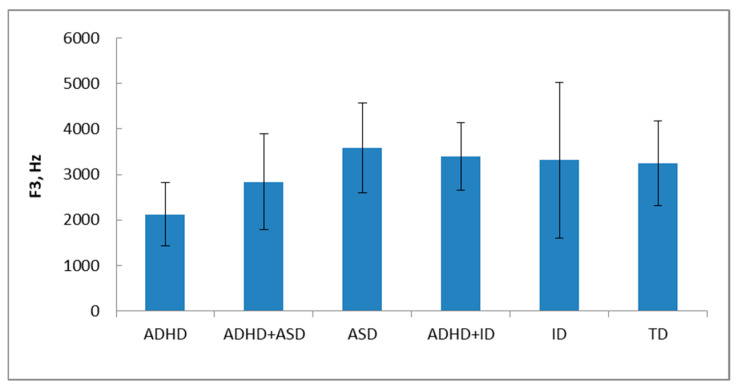
F3 values for 6 groups of children (Average ± standard deviation). Vertical axis –F3 values, Hz; horizontal axis—groups of children.

The F3 values of stress vowels of ADHD children are significantly lower (*p* < 0.0001) compared to the corresponding features of other groups of children (Figure 4, Table 7). The F3 values for children with ASD are maximal and significantly higher vs. F3 values of TD children (*p* < 0.001), children with ADHD+ASD (*p* < 0.0001), and ID (*p* < 0.05) (Figure 4).

**Figure 5:**
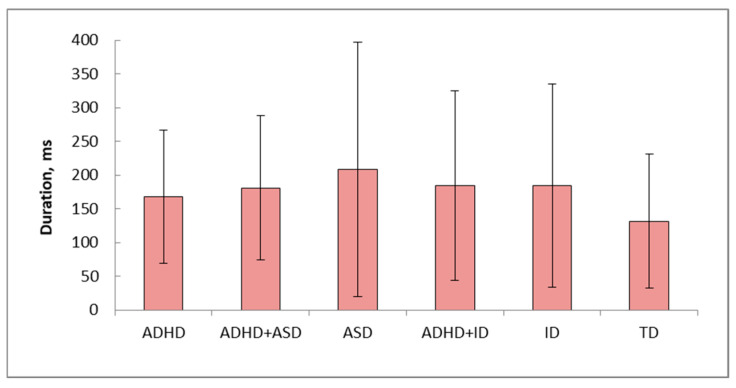
The stressed vowels’ duration for 6 groups of children (Average ± standard deviation). Vertical axis—duration, mc; horizontal axis—groups of children.

Groups of children with ADHD and TD children have minimal values of duration of stressed vowels in words and do not differ significantly (Table 7). Differences in stress vowel duration between ADHD+ID and ID (*p* < 0.05), ADHD+ID and ASD (*p* < 0.005), ASD and ID (*p* < 0.0001) were revealed (Figure 5, Table 7).

Original data on the acoustic features of speech are presented in the Supplementary Materials.

The links between children’s ages and acoustic features of speech are shown (Table 8). Children with ADHD, ID, and ADHD+ASD at a younger age had higher pitch values. Young children (5–7 years old) with ADHD have higher values of F1 and F2, ADHD+ID children—values of F2 and F3, and ID children—values of F2. Children with ASD had longer vowel durations in words at a younger age. The revealed influence of age on the pitch values and formant frequencies reflects natural age changes in the structure of the vocal tract.

Young children with ASD have higher scores on the CARS scale, which reflects the severity of autistic disorders F(1, 364) = 43.88 *p* < 0.00001 (R^2^ = 0.108 β = −0.328)—regression analysis. Young children (5–7 years old) with ADHD+ASD have higher scores on the CARS scale F(1, 276) = 114.34 *p* < 0.0001 (R^2^ = 0.293 β = −0.541).

The speech of children with ADHD is characterized by low pitch values and the third formant, values of the range between the first two formants, and high values of the VAI. The influence of children’s age on these acoustic characteristics of their speech was not revealed. Children with ADHD have low pitch values, high values of the VAI, and the shortest duration of stressed vowels in words compared to the corresponding data for children with ADHD+ASD, ADHD+ID, ID, and ASD, but these acoustical features do not differ significantly from the speech features of TD children. The speech of children with combined disorders—ADHD and ASD—is characterized by maximal values of pitch (high values for all ages), while children with ADHD and ID have low values of the VAI, reflecting unclear pronunciation of words [49]. Comparing the ADHD and TD groups, ADHD and ADHD with comorbidity (ADHD+ASD and ADHD+ID) at the participant level confirmed the results obtained for utterances/words and vowels.

## 4. Discussion

Our study aimed to find the peculiarities of children with ADHD based on behavioral characteristics and acoustic features of speech. We used a complex approach to study children with ADHD—expert analysis of children’s behavior, acoustic spectrographic analysis of children’s speech and questionnaires about early child development filled out by parents. We clearly separated the groups of children with ADHD and ADHD with comorbidity and selected controls. The small number of children in each group was a consequence of this division. Our study was exploratory, as there are very few studies that examine ADHD speech features. For Russian-speaking children with ADHD, there are no data except our pilot study [23] about behavior and speech in the scope of one approach and one task.

All children were tested while performing the “co-op play” task of the CEDM, since this task makes it possible to assess the specifics of behavior, play activity, and receive speech from children. Games and toys have a very important role in children’s lives. Playing is an action involving fun and learning in which a child willingly participates, while toys are tools they use while performing these actions [53]. It contributes to the development of cognitive, motor, psychosocial, emotional, and linguistic skills [54]. “Co-op play” is the act of assuming a role or character in a fictional world, making decisions about the character’s actions based on the rules. The “Birthday” “co-op play” used in our study allows us to obtain behavioral and speech data and identify ADHD symptoms based on voice, speech, and behavioral characteristics, such as pronunciation patterns, impulsivity, inattention, and failure to follow the rules of the game—the order of actions and lines. Moreover, comparing children with ADHD and children from other groups highlights the specificity of children with ADHD. The play situation, lasting 2 to 5 min, allowed for the collection of a variety of valid material from different groups of children using non-invasive methods.

The characteristics of behavior and acoustic features of speech of children with ADHD and ADHD and comorbidity when performing the “co-op play” test task were obtained and explored. Children with ADHD had lower scores for play and behavior than TD children, with the greatest differences for characteristics of play, “Playing for toy”, and behavior, “Displaced activity” and “Losing attention”. The study hypothesis was supported. On the one hand, such a result was expected, since these behavior indicators are included in the diagnostic characteristics of the DSM-V [2] and were chosen for the analysis of the “co-op play”, on the other hand, they indicate the legitimacy of using the selected test task, they are supported by other research [6,55] and supplement them with a description of the characteristics of play activities of children with ADHD. In contrast to identifying these characteristics based on the diagnostic interview data, in our study, they were determined based on a more detailed analysis, expert analysis of play video recordings using two approaches.

For the test task “co-op play”, children with ADHD+ASD, ADHD+ID received lower scores than children with ADHD, which was shown in other studies—the subgroups of subjects with ADHD and related disorders have worse outcomes, as evidenced by significantly greater social, emotional, and psychological difficulties [56]. Thus, a comparison of the behavioral characteristics of children with ADHD and children with ADHD and comorbidities revealed differences between them. This confirms the first hypothesis.

An analysis of the acoustic features of speech, conducted with a selection of those characteristics that were previously identified in total as markers of ASD, ID, and DS [35,36,37,38,39,40,41,42] revealed specific characteristics for ADHD and ADHD with comorbidity.

Children with ADHD did not significantly differ from TD children by pitch values of speech, which has also been shown by other researchers [22,23], indicating that the vocal behavior in children with ADHD is different from that of controls [21,22].

The speech of children with ADHD is characterized by articulation peculiarities—low values of the third formant and the difference between the first two formants, compared to the corresponding features of children from other groups. These acoustic characteristics explain the peculiarities of the timbre characteristics of the voice noted by other researchers [21,22]. Children with ADHD are characterized by low pitch values, high values of vowel articulation index, and the shortest duration of stressed vowels in words compared to children with developmental disorders, but these indicators do not differ significantly from the corresponding speech features of TD children. The speech of children with ADHD+ASD is characterized by maximal pitch values (high-pitched voice), while children with ADHD+ID have low VAI values, reflecting unclear pronunciation of words. Thus, we confirmed the second hypothesis.

The acoustic features of speech in children with ADHD and ADHD with comorbid disorders in our study were obtained for the first time using Russian-language material.

The characteristics of behavior and speech features of children with ASD and ID were considered as controls for children with comorbidity—ADHD+ASD and ADHD+ID. For children with ADHD+ASD, speech and behavior violations were more profound. According to the analyzed parameters, these children significantly differ from those with ADHD, but less so from children with ASD. The issue of identifying differences between ADHD+ASD and ASD is currently relevant, since children with ASD have a high incidence of ADHD symptoms and vice versa [57].

The stated goal of the study—to identify the behavioral characteristics and acoustic features of speech, specific to children with ADHD and ADHD with comorbidity—ADHD+ASD, ADHD+ID—in the frame of one test task, was also achieved. We presented preliminary data from the exploratory study. However, with a larger sample and confirmation of the statistical patterns identified, our findings open up broad prospects for further study of children’s speech and behavior to more accurately diagnose ADHD.

## 5. Conclusions

Based on the analysis of behavior and speech of children with TD, ADHD, and ADHD with comorbidity performing the “co-op play” test task, the set of characteristics specific to ADHD was identified.

It was shown that groups of children with ADHD, ADHD and comorbidity, and control groups differ in the characteristics of behavior associated with the development of play, play for a toy, aimless motor activity, and losing attention to play.

The speech of children with ADHD is characterized by articulation peculiarities—low values of the third formant and the difference between the first two formants, comparing to the corresponding features of TD children, children with ADHD+ASD, ADHD+ID, children with ASD, and ID. Comparison of the acoustic features of speech in children with ADHD and ADHD with comorbidity revealed that children with ADHD are characterized by low pitch values, high values of vowel articulation index, the shortest duration of stressed vowels in words compared to children with atypical development. The speech of children with ADHD+ASD is characterized by maximum pitch values, and that of children with ADHD+ID by low vowel articulation index values.

**Limitation:** A limited number of acoustical features of speech were used in this study; a small sample of children with ADHD; and a lack of separation by the leading diagnosis, predominance of attention deficit or hyperactivity. At this stage of the exploratory study, small and unequal group sizes constrain statistical power and subgroup interpretation.

In future work, we plan to increase the sample of children with ADHD and to analyze a larger number of acoustic features—values of intensity, speed of speech, duration of pauses between words and phrases in a statement.

Our exploratory study was conducted on material from a cultural-linguistic context and on a limited sample; with an increase in the sample, the availability of data for other cultural-linguistic environments and their comparability, it would be possible to discuss new diagnostic criteria.

## Figures and Tables

**Table 1:** Distribution of children with ADHD by gender, %.

Group of Children	Boys	Girls
ADHD	76%	24%
ADHD+ID	94%	6%
ADHD+ASD	90%	10%

**Table 2:** The correlation between the group of children and the characteristics of early development of children—multiple regression analysis (Forward).

R^2^	F	Independent Variable	β	SE β	B	SE B	t (54)	*p*
Dependent variable: Group of children	F(2, 54) = 10.878	Speech development	0.395	0.119	0.446	0.134	3.319	0.002
		Age of first words	0.274	0.119	0.161	0.07	2.299	0.025

R^2^—correlation coefficient (R) squared; SE—standard error; β—standardized; B—unstandardized regression coefficients; *p*—is a number describing how likely it is that data would have occurred under the null hypothesis of a statistical test. F(2, 54)—the number of degrees of freedom (2 and 54) is in brackets.

**Table 3:** Information about children participating in the study.

Group of Children	Number of Children	Boys	Girls	Age
Study 1				
ADHD	10	8	2	8.8 ± 2.9
ADHD + ASD	10	8	2	7.4 ± 1.9
ASD	10	8	2	8.0 ± 1.2
ADHD + ID	16	13	3	7.3 ± 1.5
ID	23	15	8	8.0 ± 1.8
TD	23	13	10	7.6 ± 2.0
Study 2				
ADHD	10	8	2	8.8 ± 2.9
ADHD + ASD	10	8	2	7.4 ± 1.9
ASD	10	8	2	8.0 ± 1.3
ADHD + ID	10	7	3	7.6 ± 1.7
ID	10	7	3	8.6 ± 2.3
TD	10	5	5	8.4 ± 2.4

**Table 4:** (**A**). Developmental characteristics of children with ADHD, ADHD+ASD, ASD, ADHD+ID, ID and TD according to scores of questionnaires completed by parents. (**B**). Characteristics of early development of children with ADHD, ADHD+ASD, ASD, ADHD+ID, ID and TD.

(**A**)							
**Groups of Children**		**Gestation**		**Early Development**		**Behavior and Health**	
ADHD		11 ± 2.9		31.2 ± 4.7		17.5 ± 1.9	
ADHD + ASD		12.3 ± 1.3		31.2 ± 2.0		18.4 ± 2.3	
ASD		12.1 ± 1.9		32.6 ± 4.2		17.8 ± 2.4	
ADHD + ID		11.9 ± 2.2		31.9 ± 3.7		17.6 ± 1.6	
ID		11.2 ± 1.8		30.9 ± 4.2		18.3 ± 2.7	
TD		11.4 ± 1.8		26.2 ± 3.3		16.0 ± 2.0	
(**B**)							
**Parameters**		**ADHD**	**ADHD +** **ASD**	**ASD**	**ADHD +** **ID**	**ID**	**TD**
Gestation, w	Av ± std	39.3 ± 1.5	39.4 ± 1.8	40.1 ± 1.0	39 ± 2.7	39.5 ± 1.4	40.1 ± 1.6
	median	40	40	40	40	40	40.5
Birth weight, g	Av ± std	3390 ± 361	3565 ± 505	3765 ± 402	3275 ± 505	3577 ± 667	3608 ± 478
	median	3400	3605	3765	3350	3490	3580
Apgar-1	Av ± std	8 ± 0.9	8.1 ± 0.3	7.9 ± 0.6	7.7 ± 1.2	8 ± 0.6	7.8 ± 0.8
	median	8	8	8	8	8	8
Apgar-2	Av ± std	8.2 ± 0.8	8.4 ± 0.5	8.5 ± 0.7	8.2 ± 1.3	8.7 ± 0.6	8.6 ± 0.8
	median	8	8	9	8	9	9
First words, m	Av ± std	17.1 ± 8	25.5 ± 18	30 ± 21.2	19.9 ± 12.4	17.3 ± 9.4	11.2 ± 3.9
	median	15 *	16.5 **	18 ***	12 *	15 *	9
Sits without support, m	Av ± std	8.8 ± 2.1	7.6 ± 2.0	7.9 ± 2.2	7.5 ± 1.2	8.1 ± 2.0	6.4 ± 0.9
	median	9 **	7.25	6.8	8	8	6
Stands, m	Av ± std	10.7 ± 1.9	10 ± 2	9.6 ± 1.9	10.1 ± 2.1	10.6 ± 1.8	8.3 ± 1.2
	median	11 **	10.5 *	10	10	11	8
Walks, m	Av ± std	13.4 ± 3.8	12.5 ± 2.7	13 ± 2.9	12.9 ± 2.2	13.2 ± 2.1	12.2 ± 1.3
	median	13 *	12.5	12.5 *	13	13	12

Average ± standard deviation; higher scores indicate developmental disabilities. * *p* < 0.05; ** *p* < 0.005; *** *p* < 0.001 Mann–Whitney differences between TD vs. other groups of children; w—week.

**Table 5:** The correlation between the group of children and the scores for questionnaires “Behavior & Health”, characteristics of early development, and scores for “co-op play”—multiple regression analysis.

R^2^	F	Independent Variable	β	SE β	B	SE B	t (81)	*p*
Dependent variable: Group of children	F(6, 81) = 16.433	Age of first words	−0.237	0.078	−0.266	0.877	−3.035	0.003
		Scores for “Behavior & Health”	−0.249	0.081	−0.171	0.056	−3.088	0.003
		Scores for “co-op play”	0.580	0.079	0.346	0.047	7.378	0.001

R^2^—correlation coefficient (R) squared; SE—standard error; β—standardized, B—unstandardized regression coefficients; *p*—is a number describing how likely it is that data would have occurred under the null hypothesis of statistical test.

**Table 6:** The correlation between the group of children and the scores for “Play” and “Behavior”, multiple regression analysis (Forward).

R^2^	F	Independent Variable	β	SE β	B	SE B	t (87)	*p*
Dependent variable: Group of children	F(4, 87) = 23.798	Overall scores for “co-op play”	−0.533	0.091	−0.318	0.054	−5.851	0.00001
		Develops play	−0.367	0.15	−0.79	0.322	−2.452	0.016
		Playing for toy	0.408	0.151	0.926	0.344	2.695	0.008
Dependent variable: Group of children	F(2, 89) = 39.57	Losing attention	−0.482	0.103	−1.227	0.261	−4.699	0.00001
		Displaced activity	−0.265	0.103	−0.519	0.201	−2.586	0.011

R^2^—correlation coefficient (R) squared; SE—standard error; β—standardized, B—unstandardized regression coefficients; *p*—is a number describing how likely it is that data would have occurred under the null hypothesis of statistical test.

**Table 7:** Differences in the acoustic features of speech of children in 6 groups, Mann–Whitney U Test (*p* < 0.05).

Features	Compared Groups of Children	Z	*p*-Level	r
Pitch values (F0): utterances/ words	ADHD+ASD/TD	9.873	0.000001	0.501
	ADHD+ID/TD	4.129	0.000036	0.189
	ADHD/ADHD+ID	−5.359	0.0000001	−0.237
	ADHD+ID/ID	2.158	0.030947	0.096
	ASD/TD	7.005	0.0000001	0.334
	ID/TD	2.578	0.009928	0.126
vowels	ADHD+ASD/TD	9.970	0.0000001	0.396
	ADHD+ID/TD	3.535	0.000408	0.131
	ADHD+ID/ID	2.972	0.002960	0.121
	ADHD/ADHD+ID	−3.759	0.000171	−0.155
	ASD/TD	8.874	0.0000001	0.336
	ASD/ID	7.256	0.0000001	0.300
stationary part of the stressed vowels	ADHD+ASD/TD	9.969	0.0000001	0.396
	ADHD+ID/ID	2.451	0.014250	0.100
	ASD/TD	8.969	0.0000001	0.339
	ASD/ID	−8.907	0.0000001	−0.368
Range [F2 − F1]	ADHD/TD	−9.496	0.0000001	−0.360
	ADHD+ASD/TD	−5.218	0.0000001	−0.207
	ADHD/ADHD+ASD	−3.629	0.0002851	−0.163
	ADHD/ASD	−9.626	0.0000001	−0.410
	ADHD/ADHD+ID	−8.367	0.0000001	−0.348
	ADHD/ID	−5.743	0.0000001	−0.249
	ID/TD	−3.962	0.000074	−0.153
F3	ADHD/TD	−14.617	0.001	−0.576
	ADHD/ADHD+ASD	−8.629	0.0000001	−0.406
	ADHD/ASD	−15.571	0.001	−0.690
	ADHD/ADHD+ID	−15.005	0.001	−0.657
	ADHD/ID	−14.212	0.001	−0.636
	ADHD+ASD/TD	−5.689	0.0000001	−0.229
	ADHD+ASD/ASD	−8.175	0.0000001	−0.372
	ASD/TD	4.270	0.000020	0.165
	ADHD+ID/TD	3.194	0.001405	0.122
	ASD/ID	3.018	0.002540	0.131
Vowels duration	ADHD+ASD/TD	8.046	0.0000001	0.319
	ADHD+ID/TD	7.863	0.0000001	0.299
	ASD/TD	10.396	0.0000001	0.393
	ID/TD	6.541	0.000000	0.244
	ASD/ID	3.596	0.000324	0.149

**Table 8:** Links between the age of children and acoustic features of speech (regression analysis).

Group of Children	Characteristics		*p*	R^2^	β
TD	F0 utter/word	F(1, 193) = 9.74	0.002	0.048	−0.219
	F0 vowel	F(1, 415) = 13.56	0.0002	0.316	−0.178
	F0 stat. part vowel	F(1, 415) = 17.37	0.00003	0.040	−0.200
	F1 values	F(1, 415) = 37.31	0.00001	0.082	−0.287
	F2 values	F(1, 415) = 17.60	0.00001	0.407	−0.202
	E3/E0	F(1, 400) = 8.87	0.003	0.217	−0.147
	Vowel duration	F(1, 415) = 29.01	0.00001	0.065	−0.256
ADHD	F0 utter/word	F(1, 223) =59.72	0.00001	0.211	−0.460
	F0 vowel	F(1, 280) = 53.47	0.00001	0.160	−0.400
	F0 stat. part vowel	F(1, 279) = 49.61	0.00001	0.151	−0.389
	F1 values	F(1, 279) = 7.95	0.005	0.028	−0.166
	F2 values	F(1, 279) = 7.24	0.007	0.025	−0.159
ASD	Vowel duration	F(1, 282) = 6.470	0.01	0.022	−0.328
ADHD+ASD	F0 utter/word	F(1, 192) = 30.78	0.0001	0.138	−0.372
	F0 vowel	F(1, 216) = 21.34	0.000001	0.090	−0.300
	F0 stat. part vowel	F(1, 216) = 18.91	0.000001	0.080	−0.284
ADHD+ID	F2 values	F(1, 299) = 4.41	0.03	0.121	−0.121
	F3 values	F(1, 279) = 9.51	0.002	0.033	−0.182
ID	F0 utter/word	F(1, 222) = 12.99	0.00001	0.055	−0.235
	F0 vowel	F(1, 300) = 30.78	0.00001	0.093	−0.305
	F0 stat. part vowel	F(1, 299) = 122.88	0.00001	0.071	−0.267
	F2 values	F(1, 299) = 6.75	0.009	0.022	0.149

R^2^—correlation coefficient (R) squared; β—standardized regression coefficients, *p*—is a number describing how likely it is that data would have occurred under the null hypothesis of statistical test.

## Data Availability

Data supporting the findings of this study are available upon reasonable request, subject to the regulatory requirements of the Ethics Committee of St. Petersburg University.

## Supplementary Materials

The following supporting information can be downloaded at: https://www.mdpi.com/article/10.3390/healthcare14060814/s1. Table S1: Analysis of video; Table S2: Acoustic features of speech; Text S1: Instruction for experts.

## Abbreviations

The following abbreviations are used in this manuscript:

ADHD	Attention deficit/hyperactivity disorder
ASD	Autism spectrum disorder
TD	Typically developing
ID	Intellectual disabilities
DS	Down syndrome
VAI	Vowel articulation index
CEDM	Child Emotional Development Method
DSM-V	Diagnostic and Statistical Manual of Mental Disorders 5th edition
CARS	Childhood Autism Rating Scale

## References

[1] Riglin L., Leppert B., Langley K., Thapar A.K., O’Donovan M.C., Smith G.D., Stergiakouli E., Tilling K., Thapar A. (2021). Investigating attention-deficit hyperactivity disorder and autism spectrum disorder traits in the general population: What happens in adult life?. J. Child Psychol. Psychiatry.

[2] American Psychiatric Association (2013). Diagnostic and Statistical Manual of Mental Disorders, Fifth Edition (DSM-5).

[3] Quinn B.P. (1999). Diagnostic and Statistical Manual of Mental Disorders, Fourth Edition, Primary Care Version. Prim. Care Companion J. Clin. Psychiatry.

[4] Magnus W., Anilkumar A.C., Shaban K. (2023). Attention Deficit Hyperactivity Disorder.

[5] Cortese S., Kelly C., Chabernaud C., Proal E., Di Martino A., Milham M.P., Castellanos F.X. (2012). Toward systems neuroscience of ADHD: A meta-analysis of 55 fMRI studies. Am. J. Psychiatry.

[6] Leffa D.T., Caye A., Rohde L.A. (2022). ADHD in Children and Adults: Diagnosis and Prognosis. Curr. Top. Behav. Neurosci..

[7] Antshel K.M., Russo N. (2019). Autism Spectrum Disorders and ADHD: Overlapping Phenomenology, Diagnostic Issues, and Treatment Considerations. Curr. Psychiatry Rep..

[8] Xia W., Shen L., Zhang J. (2015). Comorbid anxiety and depression in school-aged children with attention deficit hyperactivity disorder (ADHD) and self-reported symptoms of ADHD, anxiety, and depression among parents of school-aged children with and without ADHD. Shanghai Arch. Psychiatry.

[9] Ogundele M.O., Ayyash H.F. (2023). ADHD in children and adolescents: Review of current practice of non-pharmacological and behavioural management. AIMS Public Health.

[10] Sivagnanamurthi K., Govind K. (2024). Behaviour Problems of Children with ADHD. Int. J. Sci. Res. Arch..

[11] Parks K.M.A., Hannah K.E., Moreau C.N., Brainin L., Joanisse M.F. (2023). Language abilities in children and adolescents with DLD and ADHD: A scoping review. J. Commun. Disord..

[12] Dahash A.H., Mohammed S.H. (2024). Attention deficit hyperactivity disorder among children with speech difficulties. South East. Eur. J. Public Heal..

[13] Brancati G.E., Magnesa A., Acierno D., Carli M., De Rosa U., Froli A., Gemignani S., Ventura L., Weiss F., Perugi G. (2024). Current nonstimulant medications for adults with attention-deficit/hyperactivity disorder. Expert Rev. Neurother..

[14] Soler-Gutiérrez A.M., Sánchez-Carmona A.J., Albert J., Hinojosa J.A., Cortese S., Bellato A., Mayas J. (2025). Emotion processing difficulties in ADHD: A Bayesian meta-analysis study. Eur. Child Adolesc. Psychiatry.

[15] Dennis M., Krasner A., Shoulberg E.K., Hoza B., Scott H., Martin C.P. (2023). Language Problems and ADHD Behaviors: Unique and Interactive Associations with School Readiness in a Socioeconomically Disadvantaged Preschool Sample. Child Psychiatry Hum. Dev..

[16] Kats-Gold I., Besser A., Priel B. (2007). The role of simple emotion recognition skills among school aged boys at risk of ADHD. J. Abnorm. Child Psychol..

[17] Cornell H.R., Lin T.T., Anderson J.A. (2018). A systematic review of play-based interventions for students with ADHD: Implications for school-based occupational therapists. J. Occup. Ther. Sch. Early Interv..

[18] Morris S., Sheen J., Ling M., Foley D., Sciberras E. (2021). Interventions for adolescents with ADHD to improve peer social functioning: A systematic review and meta-analysis. J. Atten. Disord..

[19] Helland W.A., Posserud M.-B., Helland T., Heimann M., Lundervold A.J. (2016). Language Impairments in Children with ADHD and in Children with Reading Disorder. J. Atten. Disord..

[20] Congologlu M.A., Turkbay T., Ciyiltepe M., Durukan I., Dogangun B., Yuce M. (2009). Immediate Effects of Methylphenidate on Vocal Acoustic Parameters in Children with Attention Deficit Hyperactivity Disorder. Bull. Clin. Psychopharmacol..

[21] Garcia-Real T., Diaz-Roman T.M., Garcia-Martinez V., Vieiro-Iglesias P. (2013). Clinical and acoustic vocal profile in children with attention deficit hyperactivity disorder. J. Voice.

[22] Hamdan A.L., Deeb R., Sibai A., Rameh C., Rifai H., Fayyad J. (2009). Vocal characteristics in children with attention deficit hyperactivity disorder. J. Voice.

[23] Lyakso E., Frolova O., Matveev A., Shabanov P., Lebedev A., Nikolaev A., Kleshnev E., Grechanyi S., Nersisson R. (2026). Attention Deficit Hyperactivity Disorder: Identifying Approaches for Early Diagnosis, a Pilot Study.

[24] Goodman R. (1997). The Strengths and Difficulties Questionnaire: A research note. J. Child Psychol. Psychiatry.

[25] Kakourou N., Papaeliou C., Maniadaki K., Karaba R. Speech problems in pre-schoolers and early identification of ADHD. Proceedings of the 18th Biennial Meeting of the International Society for the Study of Behavioural Development.

[26] Vaquerizo-Madrid J., Estévez-Díaz F., Díaz-Maíllo I. (2006). A review of the alert and psycholinguistic intervention model in attention deficit hyperactivity disorder. Rev. Neurol..

[27] Jepsen I.B., Hougaard E., Matthiesen S.T., Lambek R. (2022). A systematic review and meta-analysis of narrative language abilities in children with Attention-Deficit/Hyperactivity Disorder. Res. Child Adolesc. Psychopathol..

[28] Carruthers S., Taylor L., Sadiq H., Tripp G. (2022). The profile of pragmatic language impairments in children with ADHD: A systematic review. Dev. Psychopathol..

[29] Katsarou D.V., Efthymiou E., Kougioumtzis G.A., Sofologi M., Theodoratou M. (2024). Identifying Language Development in Children with ADHD: Differential Challenges, Interventions, and Collaborative Strategies. Children.

[30] Parsons L.Q., Cordier R., Munro N., Joosten A., Speyer R. (2017). A systematic review of pragmatic language interventions for children with autism spectrum disorder. PLoS ONE.

[31] Green B.C., Johnson K.A., Bretherton L. (2014). Pragmatic language difficulties in children with hyperactivity and attention problems: An integrated review. Int. J. Lang. Commun. Disord..

[32] Zambrano-Sánchez E., Cortéz J.A.M., del Río Carlos Y., Moreno M.D., Cortés N.A.S., Hernández J.V., Cervantes T.E.R. (2023). Linguistic alterations in children with and without ADHD by clinical subtype evaluated with the BLOC-S-R test. Investig. Discapac..

[33] Khodeir M.S., Mohamed S.M., Abdel-Fattah Hegazi M. (2024). Language profile among Arabic-speaking children with attention deficit hyperactive disorder. Int. J. Pediatr. Otorhinolaryngol..

[34] Hawkins E., Gathercole S., Duncan A., Holmes J., The Calm Team (2016). Language Problems and ADHD Symptoms: How Specific Are the Links?. Brain Sci..

[35] Hubbard K., Trauner D.A. (2007). Intonation and emotion in autistic spectrum disorders. J. Psycholinguist. Res..

[36] Bonneh Y.S., Levanov Y., Dean-Pardo O., Lossos L., Adini Y. (2011). Abnormal speech spectrum and increased pitch variability in young autistic children. Front. Hum. Neurosci..

[37] Fusaroli R., Grossman R., Bilenberg N., Cantio C., Jepsen J.R.M., Weed E. (2022). Toward a cumulative science of vocal markers of autism: A cross-linguistic meta-analysis-based investigation of acoustic markers in American and Danish autistic children. Autism Res..

[38] Fusaroli R., Lambrechts A., Bang D., Bowler D.M., Gaigg S.B. (2017). Is voice a marker for Autism spectrum disorder? A systematic review and meta-analysis. Autism Res..

[39] Lyakso E., Frolova O., Karpov A. A New Method for Collection and Annotation of Speech Data of Atypically Developing Children. Proceedings of the 2018 International Conference on Sensor Networks and Signal Processing (SNSP).

[40] Lyakso E., Frolova O., Grigorev A., Gorodnyi V., Nikolaev A., Kurazhova A. (2020). Speech Features of 13–15 Year-Old Children with Autism Spectrum Disorders.

[41] Frolova O., Nikolaev A., Grave P., Lyakso E. Speech Features of Children with Mild Intellectual Disabilities. Proceedings of the Companion Publication of the 25th International Conference on Multimodal Interaction (ICMI’23 Companion).

[42] Vorperian H.K., Kent R.D., Lee Y., Buhr K.A. (2023). Vowel Production in Children and Adults with Down Syndrome: Fundamental and Formant Frequencies of the Corner Vowels. J. Speech Lang. Hear. Res..

[43] Olah J., Diederen K., Gibbs-Dean T., Kempton M.J., Dobson R., Spencer T., Cummins N. (2023). Online speech assessment of the psychotic spectrum: Exploring the relationship between overlapping acoustic markers of schizotypy, depression and anxiety. Schizophr. Res..

[44] Lyakso E., Frolova O., Kleshnev E., Ruban N., Mekala M., Arulalan K.V. Approbation of the Child’s Emotional Development Method (CEDM). Proceedings of the Companion Publication of the 2022 International Conference on Multimodal Interaction (ICMI ‘22 Companion).

[45] Schopler E., Reichler R.J., deVellis R.F., Daly K. (1980). Toward objective classification of childhood autism: Childhood Autism Rating Scale (CARS). J. Autism Dev. Disord..

[46] Scientific Report for Grant of Russian Science Foundation N 22-45-02007. https://www.rscf.ru/project/22-45-02007/.

[47] Likert R. (1932). A technique for the measurement of attitudes. Arch. Psychol..

[48] Fant G. Vocal tract area functions of Swedish vowels and a new three-parameter model. Proceedings of the 2nd International Conference on Spoken Language Processing (ICSLP-1992).

[49] Roy N., Nissen S.L., Dromey C., Sapir S. (2009). Articulatory changes in muscle tension dysphonia: Evidence of vowel space expansion following manual circumlaryngeal therapy. J. Commun. Disord..

[50] Md Juremi N.R., Zulkifley M.A., Hussain A., Zaki W. (2017). Inter-rater reliability of actual tagged emotion categories validation using Cohen’s Kappa coefficient. J. Theor. Appl. Inf. Technol..

[51] Landis J.R., Koch G.G. (1977). The measurement of observer agreement for categorical data. Biometrics.

[52] Fleiss J.L. (1971). Measuring nominal scale agreement among many raters. Psychol. Bull..

[53] https://www.merriam-webster.com/dictionary/game.

[54] Dag N.C., Turkkan E., Kacar A., Dag H. (2021). Children’s only profession: Playing with toys. N. Clin. Istanb..

[55] Carpenter K.L.H., Davis N.O., Spanos M., Sabatos-DeVito M., Aiello R., Baranek G.T., Compton S.N., Egger H.L., Franz L., Kim S.J. (2024). Adaptive behavior in young autistic children: Associations with irritability and ADHD symptoms. J. Autism Dev. Disord..

[56] Spencer T.J. (2006). ADHD and comorbidity in childhood. J. Clin. Psychiatry.

[57] Lievore R., Crisci G., Mammarella I.C. (2025). Emotion Recognition in Children and Adolescents with ASD and ADHD: A Systematic Review. Rev. J. Autism Dev. Disord..

