# Supplementary material for: Behavior and Speech Features of Children with ADHD
Source: Healthcare (Basel). 2026 Mar 22;14(6):814. doi: 10.3390/healthcare14060814 (PMC13026788; doi:10.3390/healthcare14060814)
Supplement: Supplementary file 1 [file healthcare-14-00814-s001.zip › Text S1.pdf]

Instructions for experts

Instructions for experts are provided verbally in Russian.

You will be provided with video recordings of children's “co-op play” and questionnaires to complete.

In the questionnaire, you must indicate the corresponding number for each item based on your own experience working with children. You can watch the video multiple times.

Information about the age and psychoneurological state of children is not provided to you.
